# Correlations Among High School Students' Beliefs about Conspiracy, Authoritarianism, and Scientific Literacy

**DOI:** 10.1007/s11191-022-00380-w

**Published:** 2022-10-10

**Authors:** Nikola Synak, Nikola Šabíková, Radomír Masaryk

**Affiliations:** Institute of Applied Psychology, Comenius University, Faculty of Social and Economic Sciences, Mlynské Luhy 4, 821 05 Bratislava, Slovak Republic

## Abstract

Studies consistently show the social impact of spreading epistemologically unfounded beliefs (or ‘conspiracy beliefs’), including negative effects on public health. The present study identified correlations among epistemologically unfounded beliefs, authoritarianism, and scientific literacy in a representative sample of 303 Slovak secondary school students, using the Epistemologically Unfounded Beliefs Scale, Right-Wing Authoritarianism Scale, and Scientific Reasoning Scale. Statistical analysis confirmed significant correlations among the three variables. The findings suggest that increasing scientific literacy could simultaneously reduce authoritarianism and epistemologically unfounded beliefs in secondary school students.

## Introduction

In today’s digital world, people face vast amounts of information generated by social networks and the Internet overall, in addition to traditional outlets. This places high demands on the ability to distinguish legitimate and trustworthy information sources from those that are untrustworthy, dubious, or unfounded. Unreliable news assumes different forms, from purposeful manipulation through commercial content to inadequate editorial efforts by media outlets. Low-quality content can be the result of authors or editors’ incompetence or a relaxed attitude, an over-reliance on intuitions, blindness to cognitive biases, and imprisonment in echo chambers (Čavojová et al., 2016).

Research suggests that students may not always succeed in the art of discernment. For example, between 2015 and 2016, the Stanford History Education Group (Breakstone et al., 2018; Wineburg et al., 2016) administered 56 tasks to 7804 students in the USA. One finding was that over 80% of middle school students believed that the native advertisements on websites, identified by the words ‘sponsored content’, were real news stories. The authors also focused on high school (secondary school) students exposed to an imgur post featuring a story on Fukushima which included a photograph. Students were captivated by the photograph and relied on it to evaluate the post’s trustworthiness of the post, with nearly 40% arguing that the post provided strong evidence because it presented a pictorial account of the conditions near the power plant. The authors also asked university undergraduate students to evaluate a tweet, which only a few students noted was based on a poll conducted by a professional polling firm and explained why this could strengthen the tweet’s trustworthiness. Less than one-third of the students fully explained how political agendas of relevant actors might influence the tweet’s content. Reliance on unfounded and dubious news can not only negatively impact individuals’ decision-making abilities but also harm society, and even pose a threat to democracy (Wineburg et al., 2016). Accordingly, it is extremely important to gain deeper insight into methods for helping young people discern what information is trustworthy.

One concept that operationalises dubious news content is epistemically unfounded beliefs. Research into epistemically unfounded beliefs (popularly known as fake news, hoaxes, or conspiracy beliefs) has mostly focused on identifying variables that correlate with the tendency to believe them. For example, Fasce et al. (2020) identified right-wing authoritarianism as a predictor of pseudoscientific beliefs among adults. Wood and Gray (2019) examined the relationship between conspiracy beliefs and right-wing authoritarianism, finding that right-wing authoritarianism in an adult population correlated on different levels with various types of conspiracy theories. These studies suggest that it can be greatly challenging for people to discern whether relevant facts, unfounded information, and the representation of opinions can be considered trustworthy. Analytic thinking and scientific reasoning can help to differentiate between trustworthy and untrustworthy online messages (e.g. Čavojová & Ersoy, 2020; Pennycook et al., 2020). Analytic thinking is a process that breaks down a whole into separate parts to evaluate the parts as well as the whole, which is an important process when evaluating news messages. Scientific reasoning is considered to be a more narrow skill than analytic thinking. Scientific reasoning is a set of skills and capabilities that allow us to understand the basics of science and apply basic processes in the search for relevant evidence (Dunbar & Klahr, 2012). However, most of these studies were conducted using adult populations. Thus, the present study aimed to determine whether correlations among conspiracy beliefs, authoritarianism, and scientific literacy could be identified among high school (secondary school) students. If this relationship is observed among this age group, then challenging authoritarian beliefs and improving scientific literacy could be used as a basis for potential interventions to reduce unfounded (or conspiracy) beliefs.

## Literature Review and Hypothesis Development

### Unfounded Beliefs

Lobato et al. (2014) used the concept of epistemically unwarranted beliefs. According to the authors (Lobato et al., 2014, p. 618), paranormal and conspiracy claims quite frequently fail to measure up to the ‘totality of evidence’ relevant to their specific claims. To distinguish science from pseudoscience, the authors refer to paranormal beliefs, conspiracy theory, and pseudoscience beliefs collectively as epistemically unwarranted.

Many researchers prefer the term conspiracy beliefs. Definitions of conspiracy theories and beliefs can be found in many relevant sources (e.g. Abalakina-Paap et al., 1999; Jolley & Douglas, 2017) as well as in some Slovak studies (e.g. Bahna, 2015; Panczová, 2017). These authors mostly agree that the basic ingredient of any conspiracy theory is unfounded information related to current events or various other phenomena which presents them as the product of a secret conspiracy intended to harm or control the public. Conspiracy theories differ geographically and are usually based on a country’s culture or history. However, some conspiracy theories are known worldwide, such as the ‘hidden truth’ behind Princess Diana’s death, the attacks on New York City’s World Trade Center, or installing microchips during vaccinations. Current research suggests a relatively high proliferation of conspiracy beliefs. According to an Insider poll of over 1000 respondents, nearly 80% of Americans believe in at least one scientifically unproven idea (Wang, 2019). Klobucký (2015) reported that almost one-half of the Slovak population believes that the world is secretly governed by powerful groups operating according to secret scenarios. In light of the present conspiracy belief renaissance, Byford (2014) returned to Michael Billig’s seminal writings on conspiracy theories published in the late 1970s and 1980s. Billig viewed conspiracy theories as the central pillar of any fascist ideology and studied their social and psychological aspects.

Conspiracy beliefs have a real impact on public health (Byford, 2014). For example, recently published studies link conspiracy beliefs and opposition to vaccinations (Hornsey et al., 2018; Jolley & Douglas, 2017). Conspiracy beliefs may also have additional negative social outcomes, such as affecting believers’ intentions to engage in the political process or their willingness to reduce their carbon footprint (Jolley & Douglas, 2014).

Pseudoscientific beliefs either repudiate scientific research or pose as actual science while contradicting real scientific evidence (Lundström & Jakobsson, 2012). Pseudoscience refers to a pool of information based on theories, presumptions, or methods that may superficially appear scientific but are not founded on scientific research (Tsai et al., 2012). Preece and Baxter (2000) noted the phenomenon of pseudoscientific news disseminated for profit by well-organised groups or popular media. Products based on pseudoscientific information include astrology, homeopathy, home therapies, and crystal therapy (Preece & Baxter, 2000).

Beliefs in paranormal phenomena are based on believing in physical, biological, and psychological phenomena that transcend basic physical and scientific laws (Lindeman & Saher, 2007). Such phenomena are commonly referred to as supernatural beliefs. These could include the belief in magic numbers, psychics, witches, ghosts, extra-terrestrials, or fantastic animals (Tobacyk, 2004).

Lewandowsky and Oberauer (2016) posited that the rejection of scientific findings is mostly driven by motivated cognition. Specifically, people tend to reject findings that threaten their core beliefs or worldview. Among the US public, denying scientific knowledge is presently more common on the right-wing side of the political spectrum than the left (Lewandowsky & Oberauer, 2016). Accordingly, the present study explored the concept of unfounded beliefs together with authoritarianism and scientific literacy.

### Authoritarianism

Altemeyer (1981) defined the concept of authoritarianism, based on the original definition developed by Adorno et al. (1950). The original concept of an authoritarian personality was based on a particular personality structure coupled with social influence, especially from family (Adorno et al., 1950). Altemeyer (1981) defined right-wing authoritarianism as obediently following traditional authorities in society. Altemeyer further characterised right-wing authoritarianism using three traits: a higher degree of authoritarian submission reflects an acceptance of established, legitimate authorities in society; a higher degree of authoritarian aggression reflects an inclination to inflict punishment that is perceived to be approved by conventional authorities; and a higher degree of conventionalism leads to a stronger acceptance of traditional social values (Altemeyer, 2006). Bhattacharya (2007) stated that Altemeyer establishes authoritarian aggression as a basis for right-wing authoritarianism. Altemeyer understands right-wing authoritarianism as the psychological aspect of authoritarianism itself. Authoritarian aggressiveness arises from a combination of generalised fear of a threatening and dangerous world and a strong belief that punitive treatment is necessary and justified towards specific stigmatised groups and those who are unconventional or simply unfamiliar. These groups are seen as a threat to traditional norms and values (Bhattacharya, 2007). Several research studies proved positive relationship between right-wing authoritarianism and beliefs in conspiracy theories. A study by Abalakina-Paap et al. (1999) showed that individuals with high level of right-wing authoritarianism are more suspectable to accepting conspiracy beliefs (Abalakina-Paap et al., 1999). A study focused on the relationship between authoritarianism and epistemically unfounded beliefs was done by Lieskovský et al. (2020). The research sample consisted of 107 participants (31 men and 76 women). Results confirmed a weak, positive, and statistically significant relationship between authoritarianism and all three dimensions of epistemically unfounded beliefs (Lieskovský et al., 2020).

### Scientific Literacy and Its Importance

Scientific thinking and scientific literacy are defined as the ability to understand scientific research methods and principles. These concepts also involve applying the methods and principles of scientific inquiry to reasoning or problem-solving situations and using the skills involved in generating, testing, and revising theories. Fully developed skills allow for reflection on the process of knowledge acquisition and change (Zimmerman, 2007). Some researchers (Evans & Durant, 1995; Jewett & Kuhn, 2016; Klahr et al., 2011) have focused on the conceptualisation of scientific thinking, development of scientific literacy, or application of the concept in STEM education. There is a local tradition of Slovak researchers focusing on this phenomenon (Bašnáková & Čavojová, 2018; Čavojová et al., 2019; Lesičková et al., 2019).

Bašnáková and Čavojová (2018) investigated the levels of scientific reasoning in researchers and high school teachers. Their work focused on scientific literacy, which involves an understanding of the basic methodological principles of the scientific method. The results suggested that high school teachers had a lower level of scientific reasoning than did active researchers. However, even academic researchers who participated in the study failed to reach the expected levels of scientific reasoning, with a mean score of 26.9 (79%) out of 34 points. Although this score may seem high, because of their professional focus, the researchers were expected to achieve almost perfect scores. The results differed mostly due to understanding methodological concepts such as response bias, control group, or double-blinding (Bašnáková & Čavojová, 2018).

Allum (2011) looked into relationships among scientific literacy, authoritarianism, and unfounded beliefs. Part of the Europeans, Science & Technology survey, data were collected in twenty-five European Union member states in Autumn 2004. Almost one thousand participants were recruited in every country. Results showed that participants who believe in the existence of ghosts perceived astrology as more scientific. Similarly, participants who agreed with authoritarian values were more acceptant of and believed in Astrology. The author also focused on the relationship between scientific knowledge and accepting Astrology and observed that 24% of European participants considered Astrology to be a field of science. Astrology scored higher in the perceived scientific ranking than Economy and finished right after Psychology. The study also showed that participants with higher level of scientific literacy were more likely to refuse Astrology as a field of science (Allum, 2011).

In 2018, a group of authors, led by one author of the present study, created and tested a program designed to help high school students familiarise themselves with the basic skills of distinguishing between trustworthy and untrustworthy news and processing text. The authors aimed to lower conspiracy beliefs in students through scientific literacy. After the intervention, the students reported significantly lower scores on authoritarianism and significantly higher scores in scientific literacy (Masaryk et al., 2018). The present study follows up on this previous work with the objective of expanding empirical knowledge to better operationalise the process of distinguishing between trustworthy and untrustworthy messages.

### Research Objectives

Our main objective was to gain insight into the explore correlations between epistemologically unfounded beliefs, authoritarianism, and scientific reasoning in a representative sample of Slovak high school students. At this age, students are already capable of independent thinking. The main instruments used were as follows: (1) the Epistemologically Unfounded Beliefs Scale, (2) the Right-Wing Authoritarianism Scale, and (3) the Scientific Reasoning Scale.

### Hypotheses

This study’s hypotheses were primarily based on exploring correlations among epistemologically unfounded beliefs, scientific reasoning, and authoritarianism. The hypotheses were developed based on our previous work (Masaryk et al., 2018; Lesičková et al., 2019).

For the first hypothesis, we followed up on the research by Čavojová et al. (2020), who found that scientific reasoning negatively correlates with generic pseudoscientific beliefs, health-related conspiracy beliefs, and COVID-19-related conspiracy.

- H1: *A higher level of scientific reasoning negatively correlates with unfounded beliefs.*
- H1a: *A higher level of abstract scientific reasoning negatively correlates with unfounded beliefs.*
- H1b: *A higher level of concrete scientific reasoning negatively correlates with unfounded beliefs.*

The second hypothesis was based on Drummond and Fischhoff (2017), who reported that general education, academic education, and scientific literacy were associated with a higher level of polarisation regarding religious and political topics. Their findings are consistent with the explanation that those who are more informed tend to interpret evidence to support their conclusions.

- H2: *A higher level of authoritarianism negatively correlates with scientific reasoning.*
- H2a: *A higher level of authoritarianism negatively correlates with abstract scientific reasoning.*
- H2b: *A higher level of authoritarianism negatively correlates with concrete scientific reasoning.*

The third hypothesis was developed on the basis of Bilewicz and Sedek (2015), who found that conspiracy beliefs tend to increase social distance and prejudice towards members of groups targeted by the conspiracies.

- H3: *A higher level of epistemologically unfounded beliefs positively correlates with authoritarianism.*

## Methods

The study was based on the correlational research design. Based on our hypotheses, we decided to apply correlational analysis to explore correlations among epistemologically unfounded beliefs, scientific literacy, and authoritarianism on the sample of Slovak high school students. We used the Epistemology Unfounded Beliefs, the Scientific Literacy Scale, and the Altemeyer Right-Wing Authoritarianism Test.

### Research Sample

Participants were invited using an online panel administered by a survey agency. The main eligibility criterion was being a student of a Slovak high school, and the sample was distributed proportionally by gender and region. The age span was pre-defined at 16 to 19. All participants were informed about the objectives and the course of the research project. Underage participants were recruited through their parents or guardians via the online panel. G*Power 3.1 with defined parameters returned the required sample size at 292. Following data collection, we excluded those participants who did not finish all items or failed the response time check.

The research sample consisted of 303 participants (145 men, 158 women) aged 16 to 19 years (mean age = 17.99 years). Regarding the age composition of our research sample (Table 1), 11.9% of the respondents were 16 years of age, 12.5% were 17 years, 39.9% were 18 years, and 35.6% were 19 years. The sample was representative for the population of high school students in the Slovak Republic. Our specific inclusion was equally represented by men and women and covered the entire territory of the Slovak Republic. The data were collected by a professional survey agency which also handled all relevant informed consent forms for underage respondents in August and September 2020. The project including all of its ethical aspects received approval by relevant program committee.

**Table 1:** Age distribution of participants

	*Frequency*	*Percent*
Age		
16	36	11.9
17	38	12.5
18	121	39.9
19	108	35.6
Total	303	100

Of the participants, 38.3% attended *Gymnázium* (academic track for those intending to study at a university), 60.1% attended vocational high schools, and 1.7% attended academies of applied arts. No participants attended sports-focused high schools or conservatoria (art track) (Table 2). The research sample covered all regions of the Slovak Republic with approximately equal distribution. Participants were invited using an online panel managed by a professional survey agency.

**Table 2:** Distribution by secondary school type

	*Frequency*	*Percent*
*Gymnázium* (academic track)	116	38.3
Vocational high school	182	60.1
Sports focused high school	0	0
Conservatorium (art track)	0	0
Academy of applied arts	5	1.7
Total	303	100

### Measurements

The Scientific Reasoning Scale was based on the research of Drummond and Fischhoff (2017), and adapted for the Slovak population by Bašnáková and Čavojová (2018). The scale is an operationalisation of scientific literacy and focuses on construct such as double-blinding, causality, control group, intervening variables, construct validity, ecological validity, random distribution, and conditional answering. It consists of two subscales that cover two different forms of scientific reasoning: abstract and concrete. The Scientific Reasoning Scale is an operationalisation of scientific literacy. We administered both forms to all students. Each form includes eight statements, to which respondents express their agreement or disagreement. An example item on the abstract form is as follows: ‘A researcher compares the efficiency of medication against a placebo (a pill which looks like a medicine but does not contain any active ingredients). If the assessment of results depends on the researcher’s subjective evaluation, the researcher should not know whether a patient has been administered the medication or placebo’. An example item from the concrete form is as follows: ‘A medical doctor had been using Medication X to cure depression. At a conference, the doctor learned about a new Medication, Y. The doctor decided to test which medication had a better effect on their patients. Half of her patients are given Medication X and the second half are given Medication Y. They both look the same. During regular inspections, the doctor asked the patients questions about their condition and mood. The doctor also evaluated the patients’ emotional state through subjective judgement. At the end of the treatment, the doctor assessed the effectiveness of both medications based on her notes. The doctor should not know which patients were given Medication X and which were administered Medication Y’*.* Total scores range between 16 and 32 points. Reliability of the abstract form reached Cronbach’s α = 0.34 and the concrete form Cronbach’s α = 0.42 with the overall result of Cronbach’s α = 0.62. For performance tests, internal consistency tends to be lower compared to other types of scales. The result of Cronbach’s alpha also depends on the number of items in the scale. Results for internal consistency are lower for both forms of scientific reasoning; however, the overall results for the entire performance tests are adequate.

Altemeyer Authoritarianism Test by Altemeyer (1981, 2006) was adapted for the Slovak population by Čavojová, Ballová-Mikušková, and Majerník (2015). Their study measured right-wing authoritarianism which is related to fascist tendencies, and the relationship with unfounded beliefs such as homeopathy or paranormal phenomena. The study was conducted on 400 participants (328 women) and the mean age was 19.84 years. Results indicate that participants scoring high on authoritarianism tend to be more attracted to conspiracy theories. The scale consists of 22 statements which respondents evaluate on a nine-point scale (1 = absolute definite disapproval, 9 = absolute definite approval). The reliability was Cronbach’s α = 0.86. An example item is as follows: ‘Our country desperately needs a mighty leader who will do what has to be done to destroy the radical new ways and sinfulness that are ruining us’. Total scores range between 20 and 180 points. Our study also identified the Cronbach’s α = 0.86. Based on our Cronbach’s alpha measure, we presume solid internal consistency.

We also collected data using the Epistemologically Unfounded Beliefs Scale (Halama, 2018), which has 18 items equally distributed across three subscales: conspiracy beliefs, pseudoscientific beliefs, and beliefs concerning the paranormal. In the phase 1, a total number of 151 participants (79 men, 72 women) were asked to write down four beliefs from every group of epistemically unfounded beliefs regardless of whether they believed them. Data from this phase were analysed using frequency content analysis, and six most frequently occurring beliefs were selected from every group. This resulted in 18 items of the instrument which were then submitted to 458 participants (238 men, 220 women); psychometric properties were analysed using descriptive and multivariate statistical methods. Respondents express approval or disapproval on a five-point scale (1 = absolute disapproval, 5 = absolute approval). An example item on conspiracy beliefs is as follows: ‘The pharmaceutical industry hides the existence of effective cancer medications to safeguard its financial profit proceeding from chemotherapy’. An example of an item on pseudoscientific beliefs is as follows: ‘Vaccination is more harmful than helpful to people’. An example of an item on paranormal beliefs is as follows: ‘Various fortune-tellers can really foresee the future’. Total scores range between 18 and 90 points. Regarding reliability in conspiracy beliefs, Cronbach’s α was 0.75; in pseudoscientific beliefs, Cronbach’s α was 0.62 and in beliefs concerning the paranormal, Cronbach’s α was 0.73. Halama (2018) reports Cronbach’s alpha (*N* = 458) for conspiracy beliefs at 0.80, for pseudo-scientific beliefs at 0.61, and for paranormal beliefs at 0.81. Based on this and our own results, we consider the internal consistency to be solid.

For all three scientific instruments, a higher score signifies a higher degree of agreement. Higher score in the Scientific Reasoning Scale signifies a higher degree of using scientific reasoning. Higher score in the Altemeyer Authoritarianism Test signifies a higher degree of subject to authority. Higher score in the Epistemologically Unfounded Beliefs Scale signifies a higher degree of subject to unfounded beliefs.

### Data Analysis

We used SPSS software 26 to analyse the collected data. Descriptive statistics was used to evaluate demographic items. The hypotheses were tested using Spearman’s correlation coefficient. To identify differences among variables, we used the non-parametric Mann–Whitney *U*-test for two independent groups, or an independent *T*-test. All data were tested for normality before the analyses. Based on the size of the research sample, we used the Kolmogorov–Smirnov test.

## Results

For the Scientific Reasoning Scale, participant scores ranged between 19 and 32 points (Mnd = 26; IQR = 4). Scores for the Epistemologically Unfounded Beliefs Scale ranged between 18 and 76 points (Mnd = 48; IQR = 18). Scores for the Altemeyer Authoritarianism Test ranged between 20 and 139 points (Mnd = 90; IQR = 29) (Table 3).

**Table 3:** Descriptive statistics and Spearman’s correlation coefficient—scientific reasoning, epistemologically unfounded beliefs, and authoritarianism

	*N*	*Min*	*Max*	*Mean*	*SD*	*IQR*	1	2	3
1. Scientific reasoning	303	19	32	25.67	2.930	4	—		
2. Epistemologically unfounded beliefs	303	18	76	46.33	11.782	18	− .366^**^	—	
3. Authoritarianism	303	20	139	85.32	23.029	29	− .366^**^	.256^**^	—

^**^Correlation is significant at the 0.01 level (2-tailed)

The correlations between scientific reasoning and epistemologically unfounded beliefs was negative, moderate, and statistically significant at *ρ* =  − 0.366, *p* < 0.001. Similarly, the relationship between scientific reasoning and authoritarianism was negative, moderate, and statistically significant at *ρ* =  − 0.366, *p* < 0.001. The relationship between epistemologically unfounded beliefs and authoritarianism was positive, weak, and statistically significant at *ρ* = 0.256, *p* < 0.001 (Table 3). These results supported H1, H2, and H3.

We also examined the significance and size of the correlations on the level of individual dimensions within our variables. There were two dimensions for scientific reasoning: abstract scientific reasoning and concrete scientific reasoning. The relationship between abstract scientific reasoning and epistemologically unfounded beliefs was negative, weak, and statistically significant at *ρ* =  − 0.273, *p* < 0.001. The relationship between concrete scientific reasoning and epistemologically unfounded beliefs was negative, medium-sized, and statistically significant at *ρ* =  − 0.384, *p* < 0.001. These results supported H1a and H1b. Additionally, regarding these two dimensions and authoritarianism, the relationship between abstract scientific reasoning and authoritarianism was negative, weak, and statistically significant at *ρ* =  − 0.285, *p* < 0.001, while the relationship between concrete scientific reasoning and authoritarianism was negative, medium-strong, and statistically significant at *ρ* =  − 0.371, *p* < 0.001 (Table 4). This supported H2a and H2b.

**Table 4:** Spearman’s correlation coefficient—two dimensions of scientific reasoning

	*Min*	*Max*	*Mdn*	IQR	1	2	3	4
1. Abstract scientific reasoning	8	16	13	2	—			
2. Concrete scientific reasoning	9	16	13	2	.582^*^	—		
3. Epistemologically unfounded beliefs	18	76	48	18	− .273^**^	− .384^**^	—	
4. Authoritarianism	20	139	90	29	− .285^**^	− .371^**^	.256^**^	—

^**^Correlation is significant at the 0.01 level (2-tailed)

The Epistemologically Unfounded Beliefs Scale contains three dimensions: conspiracy beliefs, pseudoscientific beliefs, and beliefs in paranormal phenomena. We compared how these dimensions related to scientific reasoning and authoritarianism in the present sample. The relationship between conspiracy beliefs and scientific reasoning was negative, moderate, and statistically significant at *ρ* =  − 0.326, *p* < 0.001. The relationship between conspiracy beliefs and authorities was positive, moderate, and statistically significant at *ρ* = 0.327, *p* < 0.001 (Table 5).

**Table 5:** Spearman’s correlation coefficient—“conspiracy beliefs” dimension vs. scientific literacy and authoritarianism

	*Min*	*Max*	*Mdn*	IQR	1	2	3
1. Conspiracy beliefs	6	27	16	8	—		
2. Scientific reasoning	20	139	90	29	− .326^**^	—	
3. Authoritarianism	20	139	90	29	.327^**^	− .366^**^	—

^**^Correlation is significant at the 0.01 level (2-tailed)

The pseudoscientific beliefs dimension had a negative, moderate, and statistically significant relationship with scientific reasoning at *ρ* =  − 0.384; *p* < 0.001. The relationship between this dimension and authoritarianism was positive, weak, and statistically significant at *ρ* = 0.255, *p* < 0.001 (Table 6).

**Table 6:** Spearman’s correlation coefficient—“pseudoscientific beliefs” dimension vs. scientific literacy and authoritarianism

	*Min*	*Max*	*Mdn*	IQR	1	2	3
1. Pseudoscientific Beliefs	6	27	14	5	—		
2. Scientific Reasoning	20	139	90	29	-.384^**^	—	
3. Authoritarianism	20	139	90	29	.255^**^	-.366^**^	—

^**^Correlation is significant at the 0.01 level (2-tailed)

The correlations between the paranormal beliefs dimension and scientific literacy was negative, weak, and statistically significant at *ρ* =  − 0.249, *p* < 0.001. Beliefs in paranormal phenomena and authoritarianism showed a positive, negligible, and statistically insignificant relationship at *ρ* = 0.067, *p* = 0.248 (Table 7).

**Table 7:** Spearman’s correlation coefficient—“paranormal beliefs” dimension vs. scientific reasoning and authoritarianism

	*Min*	*Max*	*Mdn*	IQR	1	2	3
1. Beliefs in paranormal phenomena	6	29	17	7	—		
2. Scientific reasoning	20	139	90	29	− .249^**^	—	
3. Authoritarianism	20	139	90	29	0.067	− .366^**^	—

^**^Correlation is significant at the 0.01 level (2-tailed)

## Discussion

The purpose of this study was to gain a better understanding of the levels of epistemologically unfounded beliefs, authoritarianism, and scientific reasoning among Slovak high school students. Statistical analyses showed that all three variables were interrelated, and all three relationships were statistically significant. We found a moderate correlation between scientific reasoning and authoritarianism. Furthermore, this correlations was negative; thus, the greater the authoritarianism, the lower the scientific reasoning. This could mean that if a respondent has a lower level of scientific reasoning, they may also have an increased risk of submitting to authoritarian leaders and vice versa. The results of the present study support the hypothesis that *a higher level of authoritarianism negatively correlates with scientific reasoning.* These results are consistent with the view that political and religious polarisation is significantly linked to beliefs related to scientific topics. In the study by Drummond and Fischhoff (2015), it was stated that for topics such as the Big Bang, stem-cell research, human evolution, and climate change, polarisation was higher among participants who scored higher on scientific reasoning (Drummond & Fischhoff, 2015).

Previous studies found that higher levels of scientific interest are related to lower levels of religious beliefs and higher levels of scientific approval (Evans & Durant, 1995). Furthermore, Kahan et al. (2012) argued that public divisions over climate change stem from not only scientific literacy but also the influence of a community or specific group with which one identifies. Thus, if the group denies global warming, the individual will also deny it (Kahan et al., 2012). Lewandowsky and Oberauer (2016) posited that the rejection of scientific findings is mostly driven by motivated cognition. Specifically, people tend to reject findings that threaten their core beliefs or worldviews, which is in line with the present study.

A moderate and negative relationship was also observed between scientific reasoning and epistemologically unfounded beliefs. We proposed that the lower the level of scientific reasoning, the higher the tendency to succumb to epistemologically unfounded beliefs. The results of the present study supported the hypothesis that *a higher level of scientific reasoning negatively correlates with unfounded beliefs.* This is consistent with Čavojová et al. (2020), who claimed that scientific reasoning correlates with generic pseudoscientific beliefs, health-related conspiracy beliefs, and COVID-19-related conspiracy beliefs (Čavojová et al., 2020). Bensley et al. (2014) made similar observations, finding that psychology students reduced their unfounded beliefs by improving their ability to distinguish between science and pseudoscience. Although unfounded beliefs may be based on personal beliefs, scientific thinking could help reduce unfounded beliefs by helping individuals achieve understanding and accept scientific information (Drummond & Fischhoff, 2015). Van Prooijen (2016) obtained similar results when comparing the relationship between education and conspiracy beliefs. He revealed three independent mediators of this relationship: belief in simple solutions to complex problems, feelings of powerlessness, and subjective social class. He concluded that the relationship between education and conspiracy beliefs cannot be reduced to a single mechanism, but is the result of a complex interplay among multiple psychological factors associated with education (van Prooijen, 2016).

We also identified a positive, weak, but statistically significant relationship between authoritarianism and unfounded beliefs. The present results support the hypothesis that *a higher level of epistemologically unfounded beliefs positively correlates with authoritarianism.* This is consistent with the findings of Abalakina-Paap et al. (1999), who identified a positive relationship between right-wing authoritarianism and belief in conspiracy theories. Similar results were reported in a study (Swami, 2012) which found a positive relationship between right-wing authoritarianism and belief in anti-Semitic conspiracy theories, but a negative relationship between authoritarianism and general conspiracy beliefs. As noted in the ‘Introduction’ section, reliance on unfounded and dubious information may have negative impact on individuals’ decision-making abilities, as well as cause harm to society and even threaten democracy (Wineburg et al., 2016). Our analysis focused on specific dimensions of epistemologically unfounded beliefs, and our findings were similar in relation to authoritarianism. Conspiracy beliefs had a positive, moderate relationship with authoritarianism; however, the relationship between pseudoscientific beliefs and authoritarianism was weak. Both relationships were statistically significant.

Furthermore, for authoritarianism, only the correlations with beliefs in paranormal phenomena were negligible and statistically insignificant. Lieskovský et al. (2020) used the same Epistemologically Unfounded Beliefs Scale as the present study and arrived at similar conclusions, although their findings differed regarding the belief in paranormal phenomena dimension. Unlike in the present study, their correlation analysis showed weak, positive, and statistically significant relationships between all three dimensions of epistemologically unfounded beliefs and authoritarianism (Lieskovský et al., 2020). Our interpretation of these findings is that the relationship between authoritarianism and pseudo-scientific beliefs, as opposed to paranormal beliefs, can rely on the basic concept of conspiracy theories in which current events or various phenomena are thought to be caused by secret conspiracies meant to harm or control the public.

We more closely examined the dimensions of epistemologically unfounded beliefs and their correlations with scientific reasoning. The pseudoscientific beliefs and conspiracy beliefs had moderate negative relationships with scientific reasoning. This pattern of results was consistent with previous literature reporting that pseudoscientific beliefs repudiate scientific research (Lundström & Jakobsson, 2012), and exposure to conspiracy theories can affect the participants’ intention to engage in political processes or their willingness to reduce their carbon footprint (Jolley & Douglas, 2014). Paranormal beliefs showed a weak relationship with scientific reasoning. Based on these results, we propose that increasing scientific reasoning may lower pseudoscientific and conspiracy beliefs on topics such as global warming, chemtrails, and vaccines causing autism. Distinguishing between information based on relevant scientific evidence and that which only emulates having a scientific basis may not be easy for high school students. Most of these students have not tried to prepare and implement a sound scientific project as part of their education. Thus, increasing scientific literacy through various activities and projects could help develop their skills in discriminating between trustworthy and untrustworthy scientific information.

Our results suggest that scientific reasoning correlates with authoritarianism and epistemologically unfounded beliefs among various high school students. However, although the present results clearly support significant correlations among epistemologically unfounded beliefs, authoritarianism, and scientific reasoning, it is appropriate to recognise several potential limitations. First, the sample lacked representation of students attending sports-focused secondary schools, conservatorium (art track) secondary schools, and academies of applied arts. To investigate the correlations between scientific literacy and epistemically unfounded beliefs, it would be beneficial to explore missing information about young adults with art focused studies. Since this is a separate category of students and this education track is very specific in its approach, a study with this group may report different results than students of general academic tracks or vocational schools.

Second, the gender distribution in the present sample was uneven. Furthermore, the length of the questionnaire could also be a limit of the study. The instrument consisted of 3 scales featuring 61 items which could have made it difficult for young adults to concentrate.

In future research, it would be useful to extend the current findings by examining insights into epistemologically unfounded beliefs, authoritarianism, and scientific reasoning using qualitative methods. Further research should also focus on causal relationships among unfounded beliefs, authoritarianism, and scientific literacy. In addition, it is necessary to further explore the role of scientific reasoning as a possible instrument to lower epistemologically unfounded beliefs and authoritarianism in an experiment involving high school students.

## Conclusion

Our study focused on the general levels of epistemologically unfounded beliefs, right-wing authoritarianism, and scientific literacy in a sample of secondary school students and the relationships among these variables. The research sample included 303 high school students, aged 16–19 years, who completed our questionnaire through an online survey panel. We used the following instruments: (1) the Epistemologically Unfounded Beliefs Scale, (2) the Right-Wing Authoritarianism Scale, and (3) the Scientific Reasoning Scale. The present research contributes to a growing body of evidence suggesting that scientific reasoning could be a useful tool to simultaneously decrease authoritarianism and epistemologically unfounded beliefs in high school students. The best results could be expected from reducing pseudoscientific beliefs, which is a dimension of epistemologically unfounded beliefs. In addition, it appears that by decreasing unfounded beliefs, it may also be possible to reduce authoritarianism.

Our research interest was closely focused on both abstract and concrete scientific reasoning. High school students achieved the best results in two items that were thematically and socially close to the world of education. We propose that creating an educational environment that fosters the development of scientific thinking should be based on learning concrete scientific procedures by means of practical examples in science and research. Such an educational environment should focus not on memorising definitions and factoids, but on the process of designing, conducting, and interpreting scientific research projects and scientific knowledge. Based on our results, we also consider it important to ensure that the training of student teachers should be based on scientific evidence. Scientific reasoning skills should be a key element of teacher training curricula on the tertiary as well as secondary level. Scientific literacy training seems to be a promising way of eliminating epistemically unfounded beliefs.

In future studies, we would like to extend our focus further and explore epistemologically unfounded beliefs, authoritarianism, and scientific reasoning through focus groups and experiments to gain deeper insight into how young people understand unfounded information. In the long term, we would like to use results from our research to design an intervention program focused on distinguishing between trustworthy and untrustworthy news. We started to develop such a curriculum in 2017, and conducted two pilot studies in high schools. In both pilot studies, for the experimental group, our intervention significantly increased scientific reasoning while simultaneously significantly reducing the authoritarianism (Masaryk et al., 2018). The results of the present correlation analysis enable us to further develop this intervention program based on real evidence. Eventually, we would like to develop a program that could be used in secondary schools both in Slovakia and internationally.

## Data Availability

The datasets generated during and analysed during the current study are available from the corresponding author on reasonable request.
